# Nocebo in Biosimilars and Generics in Neurology: A Systematic Review

**DOI:** 10.3389/fphar.2019.00809

**Published:** 2019-07-24

**Authors:** Ioanna Spanou, Theodoros Mavridis, Dimos D. Mitsikostas

**Affiliations:** 1^st^ Department of Neurology, Eginition Hospital, National and Kapodistrian University of Athens, Athens, Greece

**Keywords:** nocebo effect, nocebo, nocebo response, biosimilars, generics, neurological diseases

## Abstract

**Background:** Nocebo refers to adverse events related to patients’ negative expectations and previous experiences, mediated by several neurobiological pathways within the brain. It is common among neurological patients and affects adherence and treatment outcomes, representing a real clinical challenge.

**Methods:** We conducted a systematic search based on the PRISMA (Preferred Reporting Items for Systematic Reviews and Meta-Analyses) guidelines in MEDLINE database, using several keywords for studies that can be processed to investigate the magnitude of nocebo in generics and biosimilars used in the most common neurological diseases. The aim was to estimate its size and suggest strategies to minimize its prevalence in clinical trials and practice.

**Results:** Of a total of 2,606 identified articles, after criteria-based selection, 35 studies were included for analysis. Overall, there was vast heterogeneity across the studies concerning population, study design, and outcomes. Nocebo response could be estimated only in one double-blind randomized trial of generic glatiramer acetate in relapsing remitting multiple sclerosis that included a placebo arm. In this trial, no significant differences observed between the three arms (innovator, bioequivalent, and placebo) in favorable and unfavorable outcomes. In the open-label phase of the trial, an increased withdrawal rate was recorded in patients switched from placebo to bioequivalent (8.4%) that may be related to nocebo. In other open-label and real-world studies evaluating biosimilars or generics for brain disorders, a similar indirect nocebo effect is assuming by several investigators. Also, knowledge gaps between health-care providers and patients exist towards generics and biosimilars.

**Conclusions:** Despite its presence, the true burden of the nocebo response and effect cannot be accurately estimated in existing studies with generics and biosimilars in neurological diseases. Targeted strategies for clinical trials’ design are needed in order to measure the exact nocebo’s size.

## Introduction

### The Nocebo Phenomenon

The term nocebo (“I shall harm”) was introduced in contraposition to the term placebo (“I shall please”) by Kennedy in the early 1960s in order to distinguish the noxious from the pleasing effects of placebo (Kennedy, 1961).

In scientific literature, the terms nocebo effect and nocebo response are frequently used inaccurately as identical. Nocebo effect derives partly from patient’s negative expectation that medical treatment will harm instead of heal (Enck et al., 2008), including both specific and non-specific drug adverse events (AEs) (Benedetti et al., 2007). Nocebo response describes the side effects observed in the placebo arm of a clinical trial, and therefore, it can be measured only in presence of a placebo arm and take its full form when the trial is double-blinded (Colloca and Miller, 2011). Nowadays, nocebo phenomenon gains more attention, as it is related to lower adherence in therapy, resulting in treatment discontinuation, as well as to high rates of dropouts in clinical trials, decreasing falsely the safety of a new drug (Benedetti and Amanzio, 2011).

Nocebo is very common in neurological diseases, especially in chronic pain syndromes such as primary headaches (Mitsikostas, 2012; Mitsikostas, 2016), neuropathic pain (Papadopoulos, 2012), and fibromyalgia (Mitsikostas et al., 2012), as well as in Parkinson’s disease (PD) (Stathis et al., 2013), multiple sclerosis (MS) (Papadopoulos and Mitsikostas, 2010), and epilepsy (Zis et al., 2017). Notably, in a meta-analysis of prophylactic antimigraine randomized control trials (RCTs), almost half of the migraine sufferers reported nocebo side effects and about 5% withdrew from the study (Mitsikostas et al., 2011). Respectively in PD, nocebo side effects reported in about 65% of the patients receiving placebo and 10% of them withdrew from the study because of these side effects (Stathis et al., 2013). In a meta-analysis of MS disease-modifying treatment trials, the pooled incidence of nocebo response was 74,4% and the pooled nocebo dropout rate was 2,1% (Papadopoulos and Mitsikostas, 2010). A recent meta-analysis of placebo-controlled clinical trials in patients on antiepileptic therapy showed that 60,8% of placebo-treated patients reported at least one AE, and 4,0% of them discontinued treatment (Zis et al., 2017). The importance of studying nocebo effect/response in neurological diseases, especially those of central nervous system (CNS) and those that include pain, is the possible direct relation to this phenomenon with potential neurochemical changes within the CNS (e.g., cholecystokinergic system in nocebo hyperalgesia) that has already been studied (Colloca and Miller, 2011; Bittar and Nascimento, 2015; Carlino et al., 2016; Zis and Mitsikostas, 2018).

The nocebo effect is an important clinical challenge, especially in the era of cost-effective medicine of generic and biosimilar medications.

### Generics and Biosimilars

According to the Food and Drug Administration (FDA), a generic drug is defined as “one that is comparable to an innovator drug product in dosage form, strength, route of administration, quality, performance characteristics, and intended use” (Alfonso-Cristancho et al., 2015). Respectively, the European Medicines Agency (EMA) defines a generic drug as a “product which has the same qualitative and quantitative composition in active substances and the same pharmaceutical form as the reference medicinal product, and whose bioequivalence with the reference medicinal product has been demonstrated by appropriate bioavailability studies” (Committee for Medicinal Products for Human Use, 2010).

Biosimilars, according to FDA and EMA, are agents highly similar to an already authorized biological medicine (drug made in living cells or organisms, typically large, complex proteins), in terms of structure with no clinically significant difference in efficacy, safety, and immunogenicity, compared with the originator (Agency EM., 2005; US FOOD and Drug Administration, 2012). Biosimilars are not generics of a biological medicine, as the natural variability and more complex manufacturing of biological medicines do not allow an exact replication of their molecular micro-heterogeneity, demand specific guidelines for regulatory approval (Declerck et al., 2017), and are widely used in medicine especially in rheumatology (Dorner and Kay, 2015) and oncology (Camacho, 2017).

However, clinician’s (Cohen et al., 2017) and patient’s (Jacobs et al., 2016) concerns about safety profiles of generics and biosimilars still exist, especially when they have to switch to a generic or biosimilar drug, contributing to negative expectations and to the emergence of the nocebo effect (Rezk and Pieper, 2017). Additionally, the type of trials that are used for generics and biosimilars approval, differs from those regarding reference drugs, as the placebo arm is absent (Agency EM., 2005; Committee for Medicinal Products for Human Use, 2010), making impossible to estimate accurately nocebo’s incidence.

To our knowledge, this is the first systematic review investigating the presence of the nocebo in generics and biosimilars substitution studies in some of the most common neurological diseases, placing the emphasis upon estimating its size and suggesting strategies to minimize its prevalence in clinical trials and practice.

## Methods

### Data Sources and Search Strategy

We performed a systematic search, based on the PRISMA guidelines (Preferred Reporting Items for Systematic Reviews and Meta-Analyses, see also **Supplementary material**) (Moher et al., 2009). The MEDLINE database was used to search for related publications in the literature. The search was conducted on 13^th^ of January 2019, using different key words every time to maximize the number of possible relevant articles and minimize the loss of many due to the specificity of the subject under investigation. Search terms included: “generic AND neurology,” “bioequivalence AND neurology,” “biosimilar AND neurology,” “generic AND headache,” “generic AND Parkinson,” “generic AND multiple sclerosis,” “generic AND epilepsy,” “generic AND Alzheimer,” “bioequivalence AND headache,” “bioequivalence AND Parkinson,” “bioequivalence AND multiple sclerosis,” “bioequivalence AND epilepsy,” “bioequivalence AND Alzheimer,” “bioequivalence AND pain,” “biosimilar AND multiple sclerosis.”

The inclusion criteria were: i) studies and trials related to neurological diseases; ii) RCTs comparing brand and generic or biosimilar neurological agent, ideally including a placebo arm; iii) observational studies comparing brand *versus* generic or biosimilar neurological agent; iv) studies/surveys investigating physician’s, patient’s and pharmacist’s attitudes toward generics or/and biosimilars; and v) studies in adults (>18 years old).

The exclusion criteria were: i) studies using only one medication arm or a single-dose administration comparing brand and generic or biosimilar agent, ii) studies concerning biosimilars and generics in other medical fields (oncology, rheumatology, and psychiatry), iii) articles not published in English, iv) studies in children, and v) reviews, meta-analysis, letters, comments, expert opinions, editorials, summaries, dissertations, theses, case reports, and case series.

### Study Selection and Data Extraction

**Figure 1:**
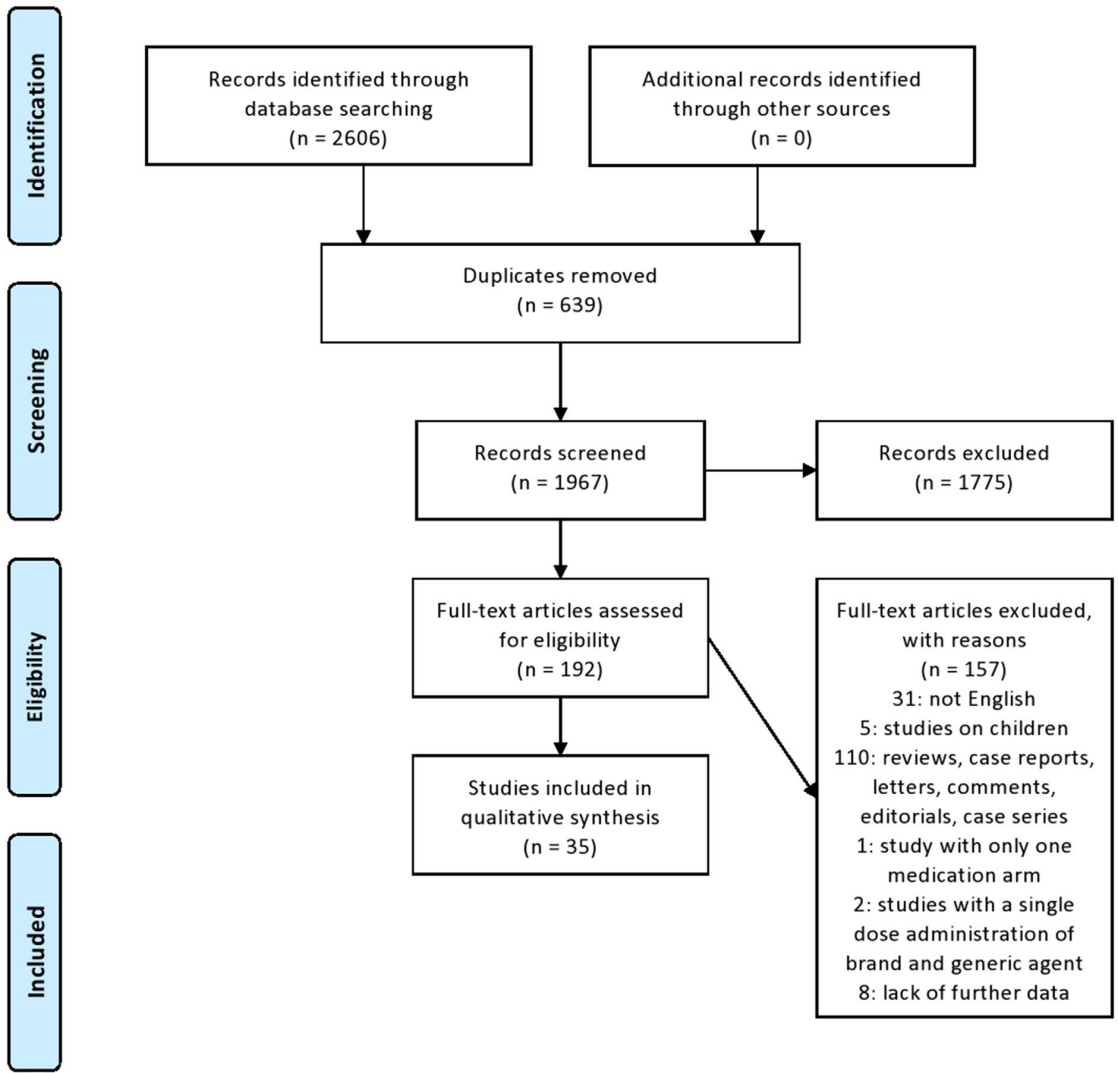
PRISMA (Preferred Reporting Items for Systematic Reviews and Meta-Analyses) flow diagram.

Two investigators (IS and TM) independently examined all titles and abstracts retrieved from the search. All full-text articles of identified abstracts that met inclusion criteria were further scrutinized. In case of disagreement during the eligibility assessment, another investigator (DDM) reviewed the abstract/full text in question and made a final objective approval. In certain cases, the corresponding authors were asked to provide relevant data. The steps of the selection process are outlined in a PRISMA flow diagram (**Figure 1**). The variables were extracted from each manuscript by applying a structured template: first author’s surname, year of publication, type of study, total number of patients and their demographics (mean age, gender), neurological disease, drug tested, number or percentage of AEs, number or percentage of dropouts due to AEs, and main findings.

### Statistical Analysis

We only performed qualitative data synthesis using our critical appraisal of individual studies and the body of evidence for each study design and identified strengths and weaknesses of each study in the discussion, without assessing publication bias. We did not attempt to perform a meta-analysis due to the heterogeneity of the study designs (randomized double-blinded studies, single-blinded studies, case–control studies, cross-sectional studies, cohort studies), populations, and results.

## Results

Of a total of 2,606 identified articles, 639 were excluded as duplicates, 1,775 were excluded after title and abstract screening because they were not related to the subject; 149 were excluded after full-text screening as not fulfilling the inclusion criteria; in particular, 31: not English language; five: studies on children; 110: reviews, meta-analysis, case reports, letters, comments, editorials, and case series; one: study using only one medication arm; and two: studies with a single-dose administration of brand and generic agent (**Figure 1**). From the remaining 43 articles, eight were excluded due to lack of further data (absence of abstract, and absent or invalid email address of the corresponding author), and finally, only 35 studies that addressed the inclusion criteria were included in this systematic review (**Tables 1**, **2**). We thus provide a narrative summary of the results, as follows.

**Table 1:** Characteristics of studies and outcomes included in the analysis.

Authors/year	Type of study	Sample characteristics (sex, age, mean ± SD)	Neurological disease	Drugs tested	Number or %AEs	Number or % dropouts due to AEs	Main findings
Cohen et al., (2015)	Randomized, double-blind (GATE)	794 p. 353 p. on generic GA F 233, 32.6 (8.6) 357 p. on brand GA F 238, 33,8 (9, 0) 84 p. placebo F 57, 32.6 (8.7)	RRMS Mean EDSS generic: 2,6 brand: 2,7 placebo: 2,7	sc GA 20 mg/day (brand/generic/placebo)	Any generic 51% Brand 54.3% Placebo 56% *Any serious* Generic 3,4% Brand GA 4,8% Placebo 2.4%	Generic 3,4% Brand 1,1% Placebo 2.4%	Mean no. of gadolinium-enhancing lesions (months 7 and 9) Generic 0,42 Brand 0.38 Placebo 0.82 Similar proportions of p. in the three groups reported AEs
Selmaj et al., (2017)	Open-label GATE extension (15-month follow-up)	728 p. Group 1: 324 p. continued generic. Group 2: 323 p. switched from brand to generic. Group 3: 81 p. switched from placebo to generic.	RRMS	sc 20 mg/day generic GA	Any Group 1 33.3% Group 2 36.5% Group 3 43.2% *Any serious* Group 1 2.5% Group 2 3.4% Group 3 3.7%	Group 1 0,6% Group 2 0,3% Group 3 8,6%	Mean no. of gadolinium-enhancing lesions (month 24) Group 1: 0.7 Group 2: 0.6 Group 3: 0.9
Abolfazli et al., (2012)	Nonrandomized observational prospective cohort	92 p. (15 excluded remained 77 for analysis)—34 on brand IFNb-1a (Avonex) F31, 30,5 ±**8.9 43 on generic IFNb-1a (CinnoVex) F31, 32.3 ± 9.0	RRMS EDSS baseline Avonex 1,9 CinnoVex 1.5	im Avonex or CinnoVex/week	NA	Increased liver enzymes: 2 p. on Avonex and 2 p. on CinnoVex	Treatment with Avonex or CinnoVex did not affect QOL during 12-month follow-up.
Nafissi et al., (2012)	Randomized, double-blind	84 p. - 60 p. completed the study (24 months) 31 on brand IFNb-1a (Avonex) F24, 33.7 ± 7.0 29 on biosimilar IFNb-1a (CinnoVex), F21, 32.2 ± 9.2	RRMS EDSS baseline Avonex 2,03 ± 1,67 CinnoVex 2.64 ± 1.12	im Avonex or CinnoVex/week	No significant differences Arthralgia oral ulcer headache and SGOT/SGPT increase higher in Avonex Skin rash and sensory loss higher in CinnoVex	Three patients (one in Avonex and two in CinnoVex) due to increase in EDSS and AEs	No significant differences between CinnoVex and Avonex in relapse, MRI lesion changes, impairment in function, and disability
Bosnyak et al., (2014)	Rater-blinded cross-over prospective	22 p. (21 completed 3-month study) M17, F5 69.3 ± 10.9	PD duration 6.5 ± 2.9 years type of PD: 17 rigid-akinetic, 5 mixed	Visit 1, 2: brand extended-release ropinirole (Requip) Visit 3,4: generic extended-release ropinirole (Ralnea)	No significant difference between brand and generic	NA	Motor symptoms, “good time”: no significant differences. Nonmotor symptoms: only the gastrointestinal section of NMSS worsening in generic but reported gastrointestinal side-effect profile was similar in generic and branded. Completion of the study: 12 p. requested brand and 9 p. generic 6 months later: 8 p. on brand and 13 p. on generic *Authors report that the patients’ preference and beliefs might have biased the results.*
Privitera et al., (2016)	Randomized double- blind cross-over	33/35 p. completed all four study periods (duration 56 days). Sequence 1: F 11,M3 42,7 (31.2 ± 55) Sequence 2: F11,M8 49,4 (32.6 ± 52.6)	Epilepsy Focal Sequence 1: 10 (71%) Sequence 2: 15 (79%) Previous history of sensitivity to switches 12% Sequence 1:1 (7%) Sequence2: 3 (16%)	All immediate-release LTG 14 p. sequence 1 (generic LTG high-generic LTG low-generic LTG high-generic LTG low) 19 p. sequence 2 (generic LMT low-generic LTG high-generic LTG low-generic LTG high)	No significant differences in seizure incidence No significant differences in AEs	None (One withdrew due to non-adherence to study protocol and one due to retinal detachment judged to be unrelated to study drug)	Bioequivalence between two disparate generic LTG products Switching between two generic products of LTG was not associated with loss of seizure control or with any change in AEs. *Authors propose a possible nocebo effect* for* the inconsistency between RCTs and patients’ concerns about generics in real world.*
Ting et al., (2015)(Duh et al., 2009)	Randomized, double-blind cross-over	34/35 p., all generic brittle, completed the protocol (2 months) M 20, F15 19–66 (42)	Epilepsy Focal 27/35	Immediate-release LTG Sequence1: generic-brand-generic-brand Sequence 2: brand-generic-brand- generic Total comedications (AEDs and non-AEDs): 3.4 (average)	Excluding 1 p.* total no. of seizures on generic and brand no difference 54 and 49, respectively No patient reported increased seizure severity Total no. of AEs during generic and brand nearly equal 14 and 15, respectively**	1/35 (Self-perceived tolerability and efficacy problems- returned to baseline 1 day after exit)	Generic LTG: bioequivalence with brand-name LTG *One subject: 267 focal motor seizures, primarily on generic, although his brand and generic pharmacokinetic profiles were identical (finally associated with increased physical activity) **One other subject: reported the 21% of all AEs, with no correlation to product or drug levels *Authors claim that therapeutic outcomes can be dominated by factors that are difficult to identify and may not be due to product’s pharmacokinetic performance (possible nocebo effect).*
Reimers et al., (2017)	Prospective nonrandomized cohort	33 p. Group 1: 17 F12, M5 Mean age 55 Group 2: 16 F9,M7 Mean age 52 Observation period before switch 10 weeks, study period 8 weeks	Epilepsy Focal: most common type for both groups Almost half of the p. seizure free at inclusion	Group 1 branded LEV Group 2 branded switched to generic LEV	NA	NA	Equal fluctuation of LEV serum concentrations with brand and generic No patient switched back to branded LEV. None of the patients that were seizure-free the year before inclusion experienced seizures while on generic. Taking patient preferences into account probably contributed to no switchbacks. *Authors report that in real life, differences in clinical effects of generic AEDs could be explained by the placebo and nocebo effects.*
Chaluvadi et al., (2011)	Retrospective chart review	245 p. F 131, 42.9 (13.8) Study period 1 year	Epilepsy Symptomatic 109/245 Cryptogenic 130/245	All compulsory switched to generic LEV. Polytherapy 158/245 (65%)	Increased AEs on generic 8/245 (3.3%) Increased seizures on generic 48/245 (19.6%)	NA	Overall switch-back 105/245 (42.9%) *Switch-back rate* Higher with higher age Higher among those who experienced increased AEs on generic (100% *versus* 40.9%) Higher among those who experienced increased AEs on brand (100% *versus* 41%) Higher among those with increased seizures on generic (100% *versus* 28%) *Authors implying a probable nocebo effect*
Fanella et al., (2017)	Prospective observational open-label	33/37 p. completed the study F23,M14 Mean age 39 6-month follow-up	Epilepsy Idiopathic generalized 18 p. Focal: 19 p. All seizure-free at least 6 months prior inclusion	All on monotherapy with branded LEV 36/37 switched voluntarily on generic LEV (Epitiram).	33/36 p.: no reported seizures or AEs	3/36 p. withdrew and switched-back 2: mood changes 1: allergic conjunctival injection	33/36 p. good clinical personal impression and continued generic Low variability of plasma levels between generic and branded LEV: was considered reassuring by the patients themselves, minimizing possible AEs related to the nocebo effect.
Gha-Hyun et al., (2018)	Retrospective cohort	148 p. M75, F 73 Mean age 46.0	Epilepsy Focal: 81.8% Before switching: Seizure-free 109/148 (73.6%)	All switched from brand to generic LEV.	NA Only data about seizure control 105/109 patients seizure-free on generic (96.3%)	NA	Overall 113/148 (76.4%) seizure-free 6 months after switching Increased seizure frequency: 7/148 (4.7%) Decreased seizure frequency: 10/148 (6.8%) *p. with reluctance to take generics were excluded from the study* *(implication of a possible nocebo effect).*
Trimboli et al., (2018)	Prospective open-label observational cohort	180 p. 125/180 switched F58, M67 40.8 ± 19.6 Follow-up: up to 4 years	Epilepsy Focal 90/125 (72%) Generalized35/125 seizure-free at inclusion (64%)	125 switched from brand to generic LEV 55/180 refused monotherapy 47%.	30/125 monotherapy with Matever: 14/59 (24%) polytherapy with Matever 16/66 (24%)	Monotherapy, Matever 8/14 Other two that switched back increased seizure frequency.	No significant difference: seizure frequency and AE before and after switching End of the study 112/125 continued on generic. No significant differences in seizure frequency and AE for p. on monotherapy with generic or brand
Bosak et al., (2017)	Retrospective electronic-database	159 p. F91/159 Mean age 34	Epilepsy Focal 83% Multiple ADEs > 90% of p.	151/159 switched from brand to generic LEV 8/159. continued on brand LEV	9/151 (6%) increased seizures AEs: 6/151 (4%) only at first follow-up visit Those who continued on brand none reported increased seizures or AEs.	2/151 switched back due to increased seizures.	Change between brand and generic LEV is generally safe. *Psychological aspects due to switching could contribute to the increased frequency of seizures in a small minority of p., implying a possible nocebo effect.*
Markoula et al., (2017)	Prospective open-label non-randomized	12 p. F5, M7 Mean age 38.4 ± 16.2 1-month follow-up	Epilepsy All focal	All switched from brand to generic LEV Multiple AEDs: 8/12	No change in seizures frequency and/or AEs	None	No significant difference in bioequivalence
Vari et al., (2016)	Prospective chart review	59 p. M29, F30 Mean age 26.1 58/59 completed the study (6-month duration)	Epilepsy Focal: 31/59 Focal with secondary generalization:12/59	All switched from brand to generic LEV (Matever) overnight monotherapy: 28/59 (47%)	NA	Switchback to brand 2/58 (3.4%) both due to increased seizure frequency and AE and both on polytherapy (1/59: lost to follow-up)	No significant differences: seizure frequency, AE during 6-month follow-up End of the study 56/58 (96.5%) continued generic LEV
Duh et al., (2009)	Retrospective observational cohort (medical and pharmacy claims data)	948 p. on topiramate F59-68% Mean age 33.7-37.5	Epilepsy	Topiramate brand generic Switching rates of eight AEDs of four non AEDs	NA	NA	92%: at least 1 prescription for branded topiramate 45%: at least 1 prescription for generic topiramate on average 1,4 versions of generic topiramate Switchback to branded: 12,5% Multiple generic topiramate use: higher utilization of other AEDs, higher hospitalization rates and higher total healthcare costs than brand AEDs lower generic substitution rate Users of generic AEDs more likely to switch back compared other chronic disease drugs.
*(Continued)*							
Mikati et al., (1992)	Randomized, double-blind cross- over	13 p. M7, F6 6-month study duration 10 completed the study	12/13 Epilepsy 1: prophylaxis (intracranial surgery) Monotherapy: all	Extended-release PHT sequence 1 brand (3 months)- generic (3 months) Sequence 2 generic (3 months)- brand (3 months)	AEs: no difference 9/10 mild, transient, tolerable (most cases not related to PHT levels) 1/10 severe: toxic levels of PHT	3/13 (increased seizures) could not be attributed to change in PHT levels (1 on brand and generic drug, 1 on generic, 1 on brand)	Generic substitution of PHT can be associated with increases in PHT serum concentrations
Bautista et al., (2011)	Prospective chart-review (questionnaire)	121 p. M44/121 Mean age 41	Epilepsy 77/121: localization-related, cryptogenic seizures 63/121: < 1 seizure/year 72/121: at least on 2 ADEs	Brand or generic AEDs 71/121 switched 13/121 uncertain	14/71 (20.6%) increased AEs 18/71 (25.7%) increased seizure frequency	NA	For those who switched: Increased seizure frequency: high baseline seizure count and high BMQ-G score Increased AEs: high BMQ-G score *High BMQ-G score reflects patient’s negative attitude toward the use of medications, with the authors implying a possible nocebo effect.*
Gagne et al., (2015)	Retrospective (electronic medical and pharmacy claims data)	19.760 initiators of AEDs F40% Mean age 75	Epilepsy 422/19.760	Initiators of AEDs (brand or generic) 18.306 (93%) generic p. with epilepsy: 48% initiated a generic	NA	NA	In the matched-cohort: initiators of generic AED: fewer adverse seizure-related clinical outcomes and longer continuous treatment periods before experiencing a gap than those who initiated brand-name version of the same AED
Kesselheim et al., (2016)	Retrospective population-based case cross-over	83,001 p. on generic AED with seizure-related hospital admission/emergency room visit Group 1: 59,344 p. with at least one same drug refill F53.51%, Mean age 34.21 Group 2: 5,200 p. with at least 1 switch F53.73%, 34.12	Hospitalization primary diagnosis Epilepsy Group 1 48,85% Group 2 68.12% Myoclonus Group 1 0.32% Group 2 0.25% Convulsions Group 1 41.25% Group 2 18.21%	59,344 p. with at least one same-drug refill 5,200 p. with at least one switch (4,310 p. different color/shape switch)	NA	NA	Risk of seizure requiring hospitalization associated with: Refill of the same drug: OR 1,08 Any switch: OR 1,09 Different color/shape switch: OR 1,11 Same color and shape switch: OR 1,00
Rahman et al., (2017)	Retrospective	NA	NA	Report of AEs of LTG, carbamazepine, oxcarbazepine for previous 11 years	LTG 27,150 71.32% brand 27.04% generic 1.64% authorized generic Carbamazepine 13,950 57.01% brand, 40.82 generic, 2.17 authorized generic Oxcarbazepine 5,077 66.36% brand, 32.46 generic, 1.18% authorized generic	NA	Brands and generics similar reporting rates after accounting for generic perception biases *“The problem of generic efficacy and tolerability could be partially psychological.” (imply a nocebo effect)* Reporting OR for suicide/suicidal ideation: higher for generic LTG and carbamazepine compared with authorized generic and brand after accounting for generic perception biases
Lang et al., (2018)	Retrospective case control	3,530 p. Seizure group 1,765 p. Seizure free group (Controls) 1,765 p. Both groups F48,9% 53.7 ± 19.8	Epilepsy	Seizure group: on generic 76.1 Seizure-free group: on generic 73% Change of manufacturer: seizure group 26.8% controls 14.2% Seizure group switched from brand to generic 5.5% (*versus* 2.4% for controls) and from generic to generic: 14.7% (*versus* 7.1% for controls)	NA	NA	In previously seizure-free p. switching the manufacturer of AED: higher risk for seizure recurrence Elderly p. may especially be at risk. *A possible factor leading to an increased risk* *for breakthrough seizures could be a nocebo effect.*

F, female; M, male; AE, adverse events; p., patients; AED, anti-epileptic drugs; NA, not available; RRMS, relapsing remitting multiple sclerosis; No, number; GA, glatiramer acetate; EDSS, expanded disability status scale; IFNb, interferon beta; QOL, quality of life; sc, subcutaneously; im, intramuscular; SGOT, serum glutamic oxaloacetic transaminase; SGPT, serum glutamic-pyruvic transaminase; f MRI, functional magnetic resonance imaging; dmPFC, dorsomedial prefrontal cortex; dlPFC, dorsolateral prefrontal cortex; PD, Parkinson’s disease; NMSS, Nonmotor Symptom Assessment Scale; LTG, lamotrigine; LEV, levetiracetam; PHT, phenytoin; BMQ-G, Beliefs About Medicines-General questionnaire; OR, odds ratio.

**Table 2:** Characteristics of studies and outcomes included in the analysis.

Authors/year	Name and type of study	Sample characteristics (sex, age, mean ± SD)	Neurological disease	Drugs	Main findings
Saposnik et al., (2018)	Web-based study prospective study	117 neurologists 90 completed the survey F42, M48 46.4 ± 10.3.	All prescribers of MS drugs 31/90 primarily focused on MS.	Primary or equal prescribers of generics: 46/90 (51%)	*Higher prescription of generics:* Older age (OR 1.19; 95% CI 1.00–1.42) General neurologist (OR 3.91; 95% CI 1.19–12.8) More willing to take risks in multiple domains (SOEP score OR 1.06; 95% CI 1.01–1.12) *Therapeutic inertia* lower for exclusively prescribers brand name compared to those who prescribe at least some generics (50.0% *versus* 79.7%)
Faasse et al., (2016)	Counterbalanced observational cohort	87 p.—81 completed four treatment conditions F83% 20.8 (3.5)	Headache	Two doses: brand label (Nurofen) Two doses: generic label In reality: half were placebo half were active ibuprofen.	*Pain reduction* Branded tablets: active or placebo did not differ. *Generic active tablets: significantly greater pain relief than generic placebo* Generic labelled placebo: significantly higher AEs *than brand name labeled placebo*.
Fehse et al., (2015)	Single-blind	30 healthy adults M: all 32 (6.39)	Thermal stimuli on forearm	Brand name acetylsalicylic acid (Aspirin) group generic acetylsalicylic acid (1A Pharma) group In reality: all placebo	Mean behavioral pain ratings decreased significantly in brand name. *f MRI* Brand name: significant bilateral activation in the dmPFC and in the dlPFC compared to generic
Piguet et al., (2015) (Selmaj et al., 2017)	Prospective qualitative, (face-to-face interviews)	25 p. F12, M13 51 (15)	Non-specific chronic musculoskeletal pain	Current generics analgesic intake 44% Intake of generics analgesic ever 80%	Majority of the p. discussed the switch with the pharmacist. Trusting the prescriber physician or pharmacist emphasized in 50% and 25% respectively Hesitation to use generics: 33%—predominantly doubts about similarity *Authors imply a probable nocebo effect.*
Gollwitzer et al., (2016)	Retrospective population-based cohort	31,317 p. F15,895 (50.8%) M15,422 (49.2%) 57.4 (19.7)	Epilepsy	Old AED New AED Brand/generic AED	Good adherence: 64.7% Good adherence: more common: New compared to old AED (OR 1.52) Branded compared to generic AED (OR 1.44*)* *Authors imply a probable nocebo effect.*
Guberman et al., (2000)	Retrospective (questionnaires)	83 p. M:38, F:42, NA:3 Mean age NA 46 Neurologists 11/46:expertise in Epilepsy	Epilepsy	Brand/generic AED	Patients Currently on generic: 20%, not sure: 22% Switched from brand to generic: 16%, not sure: 22% Unaware of the possibility of receiving generics from different manufacturer: 86% Pharmacists virtually never inform about receiving a generic Patients do not seem concerned about generic substitution *Neurologists* Unaware that a generic could be substituted by the pharmacist despite writing brand name: 22% Feeling any generic AED could be safely substituted for the brand: 30 to 67% Uncomfortable starting a generic of one of the newer AED: 55%
Ngo et al., (2013)	Retrospective (self-administered questionnaire)	700 p. invited 47 p. responded M20,F27 Mean age NA	Epilepsy Generalized 36% Not sure about epilepsy type: 34% Last seizure ≤3 months ago: 45%	Currently at least one AED: 85%	Never asked for a generic when having their prescriptions filled: 76,6% Brand substitution with generic should only be done with p. consent: 87%, and with doctor consent: 64% Concerned about effectiveness of a generic: 70% Concerned about safety of a generic and that substitution could have negative consequences: 55% Uncomfortable taking a generic AED: 68% 2 p. indicated that their neurologist advised them not to use a generic. Would use generics for acute short-term conditions 64*%* *Authors suggest further research for a probable nocebo effect*.
Berg et al., (2008a)	Retrospective online survey (questionnaires)	3,606 p. invited 500 p. responded F 55%, average age 47 6,359 physicians invited 606 physicians responded	Epilepsy well-controlled: 62% generalized 23% not sure 27% Neurologists 70% GPs26% Epileptologists 4%	AEDs brand-name/generic	*Patients* Concerned about efficacy of generic: 65% Substitution with a generic could have negative consequences: 70% Breakthrough seizures linked to generic substitution: 34% *Physicians* Concerned about an increase in breakthrough seizures when switching: 88% Neurologists more concerned than GPs Unaware that a pharmacist may substitute a branded medication for a generic without physician’s consent: 38% of p., 25% of physicians
Berg et al., (2008b)	Retrospective semi-qualitative online survey (questionnaire, case review form)	50 neurologists (included to the analysis) reporting data from 50 p. with epilepsy and breakthrough seizures	Epilepsy breakthrough seizure complex partial: 36% convulsive: 64% Prior to the breakthrough seizure: 62% of neurologists saw their p. One or two times/year Increased office visits until seizures controlled: 10% of neurologists	On branded AED for at least a year before switch: 86% On generic AED ≤ 3 months before breakthrough seizure: 78%	40% of neurologists: patient switched to a generic without their consent. 16% of neurologists: pharmacy substituted a generic without approval. 92% of neurologists switched patients back to the original AED after breakthrough seizure and 96% of these p. regained seizure control per their physician’s assessment.
Wilner, (2002)	Retrospective (questionnaire)	845 Physicians (258 from AES survey and 587 from AAN survey)	Epilepsy AES survey: 50% of physicians had >75% epilepsy p. AAN survey: 5,6% of physicians had >75%epilepsy p.	Carbamazepine AES survey: 41,9% of physicians estimated a 30% of p. on generic and 30.2% of physicians estimated a 50% of p. on generic AAN survey: 40% of physicians estimated a 30% of p. on generic and 30.2% of physicians estimated a 50% of p. on generic	AES survey: 86,4% of physicians: uncomfortable with patients receiving multiple formulations of generic carbamazepine AAN survey: 80.3% of physicians did not endorse generic substitution of carbamazepine Overall substitution rate by pharmacists: 68% (much higher than estimated by the surveyed physicians)
Crawford et al., (1996)	Retrospective (questionnaire)	40 GPs 1,343 p. replied M49.9%, F 50.1% 45.79 (20.8)	Epilepsy	Unbranded sodium valproate: 13,2% (of 39,2% on valproate) Unbranded carbamazepine: 38.4% (of 37.8% on carbamazepine) Unbranded phenytoin: 39,2% (of 32,6% on phenytoin) Switching: 18.7% (251/1,343)	*Patients* 74.5%: close interest in their medication Problems after switch: 29.5% Validated problems: 10.8% (27/251, in nine seizures and in 21 AEs)—Those patients take more frequently close interest to drug therapy compared to those with no problems *Authors imply a probable nocebo effect.*
Das et al., (2019)	Prospective observational (questionnaire)	148 p. M, F: approximately equal numbers Mean age: NA	Epilepsy Focal epilepsy: 78.4%	Brand even though generic is available: 29/148 (all of them: GB) generic: 117/148 (31 GB, 86 not GB) brand since no generic available: 2/148 (all of them: not GB) currently on a problem AED 115/148 (54/60 GB p. and 61/88 not GB p). Four total medications: 18.9%	GB: 60/148 (40,5%), negative opinion of generics: 57/60 Not GB 88/148 (59,5%) *Factors associated with GB* Currently on a problem AED On ≥6 medications Switch problem: 41/60 GB p. (32 brand-generic switch problem and 10 generic-generic switch problem) Uncontrolled seizures: GB 54/60, not GB 44/88 Formulation-specific AED AEs: GB 51/60, not GB 45/88 *Authors imply a possible nocebo effect.*
Roth et al., (2016)	Retrospective (questionnaire)	121 Pharmacists M33, F86, NA 2 35.2 ± 9.1	Epilepsy 63% of pharmacists estimated:≤10 epileptic p./month	AEDs	Total mean score of correct answers: 48 ± 15% Correct scores on the true/false statements about the risks of generic switches: 89% Longer time since training completion was associated with a lower total score

F, female; M, male; OR, odds ratio; SOEP, Socio-Economic Panel Score; f MRI, functional magnetic resonance imaging; AED, antiepileptic drug; OR, odds ratio; GPs, general practitioners; AES, American Epilepsy Society; AAN, American Academy of Neurology; GB, generic brittle.

### Studies on Biosimilars and Generics in Multiple Sclerosis

In 2012, an Iranian (Nafissi et al., 2012) randomized double-blind study of 60 patients with relapsing-remitting multiple sclerosis (RRMS) receiving either branded intramuscular interferon b-1a (IFNb-1a) or its biosimilar form demonstrated no significant differences in efficacy and safety between the two groups during the 2-year follow-up.

In the same year, another Iranian (Abolfazli et al., 2012) nonrandomized observational study of 77 patients with RRMS found that treatment with the originator intramuscular IFNb-1a or its biosimilar form did not affect the quality of life between the two groups during a 12-month follow-up.

As for the bioequivalent of the glatiramer acetate (GA) is concerned, two studies were found. Firstly, the randomized double-blind GATE study (Cohen et al., 2015) of 794 patients with RRMS, where three arms were included (353 patients on generic, 357 patients on brand name, and 82 patients on placebo), demonstrated an equivalent efficacy, safety, and tolerability between branded and generic GA. More specifically, similar proportions of patients in all three groups reported any AE during the 9-month follow-up (51% on generic, 54% on brand name, and 56% on placebo), and no statistically significant difference was found regarding the dropouts (3.4% on generic, 1.1% on brand name, and 2.4% on placebo).

The open-label extension of GATE study (Selmaj et al., 2017) included 728 patients and did not reveal an increase in the reported AEs or the discontinuation rates due to AEs between the blind and the unblinded phase for those who continued on bioequivalent and those who switched from innovator to bioequivalent during the 15-month follow-up (33.3% *versus* 36.5% and 0.6% *versus* 0.3%, respectively). On the contrary, among the group of switching from placebo to the bioequivalent GA, the 43.2% of patients reported any AE and the 8.6% withdrew from the study due to AEs.

Finally in 2018, a web-based study among 90 neurologists (Saposnik et al., 2018), all of who prescribed MS treatments, demonstrated that half of them were primary or equal prescribers of generics or biosimilar MS drugs, a fact that was associated with a higher incident risk of “therapeutic inertia.” Factors associated with higher prescription of generics were older age, being a general neurologist, and more willingness to take risks in multiple domains.

### Studies on Generics in Headache

In 2016, an observational study in Auckland (Faasse et al., 2016) in 87 undergraduate students with frequent headaches investigated the impact of drug labeling on medication effectiveness and safety. Pain reduction following the use of brand name labeled tablets was similar for active ibuprofen and placebo, while if the tablets had a generic label, placebo tablets were significantly less effective compared to active ibuprofen. Also, fewer side effects were attributed to placebo tablets with brand-name labeling compared to the placebo tablets with a generic label.

### Studies on Generics in Pain Syndromes

In 2015, a single-blinded functional magnetic resonance imaging (fMRI) study investigated the underlying brain processes mediating placebo response to a brand labeled analgesic (Aspirin) compared to a generic labeled analgesic (generic acetylsalicylic acid), on 30 healthy subjects receiving thermal stimuli on their left arm (Fehse et al., 2015), while in reality, all subjects received placebo. Mean behavioral pain ratings decreased significantly after “aspirin” administration but remained unchanged after the generic labeled analgesic. Also, subjects receiving placebo with the brand label demonstrated increased activity in the dorsolateral and dorsomedial prefrontal cortex, the areas of the brain that are known to be activated in placebo analgesia.

In 2015, in a face-to-face interview study of 25 patients with non-specific chronic musculoskeletal pain (Piguet et al., 2015), almost half of them reported currently generic analgesic intake. The majority of the patients had discussed the switch with the pharmacist and emphasized that trusting the prescriber physician or pharmacist was very important for the switch. However, 33% of the patients claimed hesitation to use generics, predominantly due to doubts about drugs’ similarity.

### Studies on Generics in Parkinson’s Disease (PD)

In 2014, in a cross-over study of 21 patients with advanced PD (Bosnyak et al., 2014) who switched from a branded to a generic extended release ropinirole, no significant differences were found regarding efficacy and safety profile, and after completion of the study, similar number of patients requested brand and generic ropinirole. Nevertheless, due to study design, authors could not exclude that patients’ preferences and beliefs might have biased their results.

### Studies on Generics in Epilepsy

In 1992, a double-blind randomized study (Mikati et al., 1992) found that generic substitution of phenytoin (PHT) could be associated with increases in PHT serum concentrations, that predominantly were asymptomatic, except from one that was associated with intolerable dose-dependent AEs. Additionally, three out of 13 patients withdrew due to increased seizures that could not be attributed to a change in PHT levels (one on brand and generic drug, one on generic, and one on brand).

In 2009, a Canadian retrospective cohort (Duh et al., 2009) reported lower generic substitution rates and higher switchback rates to branded drugs among antiepileptic drugs (AEDs) compared to other drugs used for chronic diseases (depression, hypertension, dyslipidemia). Especially for topiramate substitution, switchback rate was 12.5%, and multiple-generic use was associated with higher utilization of other AEDs, higher hospitalization rates, and higher total healthcare costs than brand use.

In the retrospective study of Chaluvadi et al., (2011), among 245 patients with epilepsy and compulsory switch to a generic levetiracetam (LEV), the switchback rate was found 42.9%. In particular, higher age, experience of increased AEs on generic LEV and previously on brand-name LEV, as well as experience of increased seizures on generic LEV were found to be significantly associated with switchback.

In 2011, an American study (Bautista et al., 2011) found that factors such as baseline seizure count and negative attitude toward medication influence patient’s perception of increased seizure frequency and AEs when switching to generic AEDs.

In 2015, a retrospective study of 19,760 initiators of five different AEDs for various conditions including epilepsy (Gagne et al., 2015) demonstrated that in the matched cohort for epilepsy diagnosis, patients who initiated a generic AED had fewer seizure-related hospitalizations and longer continuous treatment periods before experiencing a gap than those who initiated brand-name versions.

At the same year, a double-blind randomized study (Ting et al., 2015) among 35 generic brittle patients with epilepsy demonstrated that generic lamotrigine (LTG) was bioequivalent with the brand-name, with no significant differences concerning the number of seizures and AEs between branded and generic products. For the majority of patients reporting AEs, including the one patient who withdrew due to self-perceived AEs, there was no correlation between the number of dose-related AEs and drug level, and the authors suggested that “therapeutic outcomes could be dominated by factors difficult to identify, implying a possible nocebo effect.”

In 2016, the double-blind randomized EQUIGEN study (Privitera et al., 2016) found bioequivalence between two generic products of immediate-release LTG, and that switching between two generic products was not associated with decreased efficacy or tolerability. Interestingly, authors propose “a possible nocebo effect that could explain the inconsistency between the findings of randomized trials and patient’s concerns about generics in real-world.”

In 2016, an Italian prospective study of “overnight” switching of branded LEV (Vari et al., 2016) in 58 patients reported no significant differences in terms of seizure frequency and occurrence of AEs during 6-month follow-up, with a rate of switchback to brand LEV of only 3.4%.

In another retrospective population-based study of 83,001 patients on generic AEDs, seizure-related hospital admissions or emergency room visits were investigated (Kesselheim et al., 2016). The study demonstrated modest increase in risk of severe seizure in the period shortly after a refill (odds ratio 1.08), not accompanied by additional risk from switching during that refill to a different manufacturer.

In 2017, a Swedish non-randomized prospective cohort of 33 epileptic patients taking branded LEV (Reimers et al., 2017) demonstrated that in 16 of them who switched to a generic LEV, none switched back, and all seizure-free patients at the inclusion time remained stable. Furthermore, the study found equal fluctuation of LEV serum concentrations between branded and generic LEV, with the authors reporting that “in real-life differences in clinical effects of generic AEDs could be explained by the placebo and nocebo effects,” emphasizing the need to take into consideration patient’s preferences about treatment.

The same year, an Italian observational open-label study of LEV switching (Fanella et al., 2017) among 36 patients with well-controlled epilepsy, revealed that only three patients switched back due to treatment-related AEs, while the remaining patients expressed a good clinical personal impression and continued on generic. Indeed, authors reported that “patient’s awareness of the low variability of plasma levels between generic and branded LEV was reassuring and probably minimized a possible nocebo effect.”

Another substitution study of branded LEV (Bosak et al., 2017) found that the change was generally safe. Increased frequency of seizures was noted in nine patients (6%), and only two of them required switchback to the brand-name LEV. According to authors, “psychological aspects associated with switching procedure could explain the increased frequency of seizures,” implying a possible nocebo effect. AEs were noted in six other patients (4%) were mild and transient and did not lead to discontinuation or switchback.

A Greek open-label study (Markoula et al., 2017) of 12 patients switched to generic LEV demonstrated bioequivalence and no change in seizure frequency and reported AEs pre- and postsubstitution.

The study of Rahman et al., (2017) concluded that “the problem of generic efficacy and tolerability could be partially psychological,” as after accounting for generic perception biases, brands and generic AEDs demonstrated similar reporting rates for the magority of AEs.

In 2018, a Korean retrospective substitution study of LEV in 148 well-controlled epileptic patients (Gha-Hyun and Dae, 2018) did not find any significant change in seizure frequency. However, patients with reluctance to take generics were excluded from the study.

At the same year, an Italian open-label observational study (Trimboli et al., 2018) of 125 out of 180 patients who switched to a generic LEV suggested no significant difference in terms of seizure frequency and AEs before and after switching and also compared to those who refused to switch. Notably, 10 out of 125 patients stopped treatment due to AEs, and two of them switched back (1.6%), during a long follow-up period.

A retrospective German case–control study (Lang et al., 2018) found that a manufacturer switch of the same AED (brand name to generic or generic to generic) increased the risk for breakthrough seizures, especially in elderly with the authors reporting that a possible nocebo effect could explain their findings.

Overall, nine studies (Crawford et al., 1996; Guberman and Corman, 2000; Wilner, 2002; Berg et al., 2008a; Berg et al., 2008b; Ngo et al., 2013; Gollwitzer et al., 2016; Roth et al., 2016; Das et al., 2019) were found, investigating patient’s, physician’s, and pharmacist’s attitutes toward generics of AEDs.

In 1996, Crawford et al., (1996) in a study of 1,343 epileptic patients demonstrated that three out of four were closely interested in their medication, with switching problems reported approximately in one out of three. In particularly, these patients were taking more frequently close interest to their medication compared to those with no problems, with the authors implying a related possible nocebo effect.

A Canadian retrospective questionnaire survey of 83 epileptic patients and 46 neurologists (Guberman and Corman, 2000) revealed a significant unawareness of the process of generic substitution among both patients and neurologists. On the one hand, 22% of the patients were not sure if they were on a generic, and also 22% of them were not sure if they had switched from a branded AED to a generic. Moreover, 86% of the patients reported unawareness of the possibility of receiving generics from different manufacturer and that pharmacists virtually never inform them about receiving a generic. On the other hand, 22% of the neurologists reported unawareness that a generic could be substituted by the pharmacist despite writing the brand name, and that 55% of them were feeling uncomfortable about starting a generic medication of one of the newer AED.

In 2008, Berg et al., (2008a) in a retrospective online survey among 500 patients and 606 physicians found that 65% of the patients were concerned about the efficacy of a generic AED, with 70% of the patients believing that generic substitution could have negative consequences regarding their seizure control. Additionally, 88% of the physicians were concerned about an increase in breakthrough seizures when switching, with neurologists being more concerned than general practitioners. A significant percentage of patients and physicians were unaware that a pharmacist may substitute a branded medication for a generic, without physician’s consent.

A retrospective survey among 50 neurologists (Berg et al., 2008b) reporting data from 50 patients with epilepsy and breakthrough seizures revealed that 40% of neurologists mentioned that the patient switched to a generic without their consent, with 16% of them reporting that pharmacists substituted a generic without their approval. Also, 92% of the neurologists switched patients back to the original AED after the breakthrough seizure, followed by seizure control in the majority of them.

An Australian retrospective study in 47 patients with epilepsy (Ngo et al., 2013) reported that 70% of them were concerned about the effectiveness of a generic AED and were uncomfortable receiving generics to treat their epilepsy. Interestingly, two patients indicated that their neurologist advised them not to use a generic AED. However, 64% of the patients would use generics for acute short-term conditions—for example, painkillers.

In 2012, 845 physicians attending American Epilepsy Society and American Academy of Neurology meetings were surveyed (Wilner, 2002), and findings indicated that most of the physicians underestimated the number of generic substitutions that occurred from the pharmacists for brand name short-acting carbamazepine.

In 2016, a large population-based retrospective study in Germany (Gollwitzer et al., 2016) of 31,317 patients with epilepsy found good adherence on antiepileptic treatment in 65% of the patients, and that one of the factors of good adherence was the use of branded compared to a generic AED, with the authors implying a probable nocebo effect when reporting “a lack of confidence with the generics.”

A study among 121 pharmacists in Israel (Roth et al., 2016) investigating their general knowledge on treatment with AEDs demonstrated that 89% of them were knowledgeable regarding the risks of generic switches, while in other aspects, some gaps were identified, suggesting the need for better education of pharmacists regarding epilepsy and its treatment.

In 2018, Das et al., (2019) found that generic brittle patients constituted the 40.5% of the study population, with almost all of them having a negative opinion on generics. Factors associated with generic brittleness were being currently on a “problem” AED and taking at least six medications (not only AEDs). About generic brittleness physiology, authors claimed clearly a possible underlying nocebo effect.

## Discussion

In this systematic review aimed to evaluate the magnitude of nocebo in studies testing biosimilars or generics in neurological disorders, only one RCT was found, which tested the efficacy and safety of a GA bioequivalent in RRMS treatment with a 3-arm design, including a placebo (GATE study) (Cohen et al., 2015). Among patients receiving placebo, approximately 1 out of 2 reported any AE, but only 2.4% withdrew from the study due to AEs, as in other studies with innovator treatments for MS (Mitsikostas, 2016). Interestingly, the open-label extension of the GATE study (Selmaj et al., 2017) revealed an increase in discontinuation rates due to AEs among the group of switching from a placebo to the GA bioequivalent (8.6%), which could suggest that awareness of the switch may had contributed to negative expectations.

Except from the above studies, direct comparisons among double-blind and open-label or real-world studies for biosimilars and generics in neurological diseases with common denominator the probable nocebo effect were unfeasible for several reasons. Firstly, the overall number of RCTs was small compared with the number of open-label and real-world studies, and the study populations were heterogeneous. Also, the follow-up period was mainly short both in RCTs and open-label studies, which may had decreased the ability to report treatment-related AEs.

Consequently, in this systematic review, the presenting data are mainly indirectly derived from the open-label and real-world studies, and also from studies investigating physicians’ and patients’ attitudes toward these medications, with the majority of them derived from studies in epilepsy. This is the major limitation of our study.

More specifically, in nine open-label and real-world studies (Bautista et al., 2011; Chaluvadi et al., 2011; Bosnyak et al., 2014; Bosak et al., 2017; Fanella et al., 2017; Rahman et al., 2017; Reimers et al., 2017; Gha-Hyun and Dae, 2018; Lang et al., 2018), authors imply or clearly mention a probable nocebo effect associated with their results.

In five studies (Bautista et al., 2011; Bosnyak et al., 2014; Reimers et al., 2017; Rahman et al., 2017; Gha-Hyun and Dae, 2018), emphasis was given to the fact that in real life, differences in clinical effects of generics could be explained by the placebo and nocebo effects, reflecting positive or negative patient’s preferences and beliefs about generics. Furthermore, Fanella et al., (2017) mentioned that low switchback rate on branded LEV was associated with a strategy of reassuring information toward the patients about their treatment provided by the investigators, for minimizing the nocebo effect. Additionally, Chaluvadi et al., (2011) found that negative experiences while on generic or previously on branded LEV were associated with higher switchback rates, implying negative expectations and conditioning characterizing nocebo emergence. Bosak et al., (2017) reported that psychological aspects associated with switching procedure could explain the increased frequency of seizures reported in their cohort, implying a possible nocebo effect. Similarly, Lang et al., (2018) found that a probable nocebo effect could have contributed to an increased risk for breakthrough seizures, especially in the elderly after a manufacturer switch of the same AED.

Interestingly, in two substitution RCTs for generic LTG, authors indirectly mention the nocebo effect. In the first one (Ting et al., 2015), among generic brittle patients (patients with previous negative experiences with generic AED), authors suggested that therapeutic outcomes could be dominated by factors difficult to identify; as for the majority of patients reporting AEs, there was no correlation between the number of dose-related AEs and drug levels. In the second study (Privitera et al., 2016), authors tried to explain the inconsistency between the results of RCTs and real-world studies concerning generic AED substitution, proposing a probable nocebo effect.

In this review, as mentioned before, interesting findings about the nocebo effect emergence derived also from studies/surveys investigating physicians’ and patients’ attitudes toward biosimilars and generics in neurological diseases that may further explain the tendency of higher incidence of nocebo in open-label and real-world cohorts.

In particular, from the physicians’ side, five studies found clearly investigating their perceptions about generic substitution (Guberman and Corman, 2000; Wilner, 2002; Berg et al., 2008a; Berg et al., 2008b; Saposnik et al., 2018). One study concerned neurologists; all prescribers of MS drugs (Saposnik et al., 2018) demonstrated that MS specialists were more concerned about generics/biosimilars. The remaining four studies were among clinicians who treated patients with epilepsy, and revealed significant unawareness of the process of generic substitution, knowledge gaps concerning special pharmacokinetic features of AEDs, and a general level of discomfort among neurologists to prescribe generic AEDs. Indirect findings about clinicians’ concerns might emerge from a retrospective study (Duh et al., 2009) that found lower generic substitution rates and higher switchback rates to innovators, among AEDs compared to other drugs used for chronic diseases. Clinicians’ doubts about prescribed treatment may be transmitted to patients, generating new negative expectancies, thus enhancing the nocebo effect (Faasse and Petrie, 2013).

From the patients’ side, several studies investigating their attitudes toward generics use in neurological diseases were found (Crawford et al., 1996; Guberman and Corman, 2000; Berg et al., 2008a; Ngo et al., 2013; Piguet et al., 2015; Gollwitzer et al., 2016; Das et al., 2019). Patients were often unaware of the process of generic substitution (Guberman and Corman, 2000; Berg et al., 2008a; Ngo et al., 2013), while in another study, the majority of them reported that they had discussed the switch with the pharmacist (Piguet et al., 2015). Also, a significant percentage of them was not even sure if they were on a generic medication, or if they had switched from a branded to a generic AED, suggestive of their unawareness (Guberman and Corman, 2000). Furthermore, patients frequently expressed their concerns about generics efficacy and safety (Berg et al., 2008a; Ngo et al., 2013; Piguet et al., 2015), while several studies investigated the factors associated with increased switchbacks, with emphasis to patient’s characteristics, and found that a probable nocebo effect might be implicated. More specifically, patients taking more frequently close interest to drug therapy (Crawford et al., 1996), as well as patients with negative opinion of generics, currently on a problem AED and on polypharmacy (Das et al., 2019), reported higher switchback rates, with the authors suggesting an underlying nocebo effect. Similarly, better adherence was reported by patients on branded compared to generic AED (Gollwitzer et al., 2016). Finally, in one study, patients emphasized that trusting the prescriber physician or pharmacist was very important for the switch (Piguet et al., 2015).

Another interesting point of this systematic review is that drug’s cost and labeling contributes crucially to the patient’s expectations and may enhance the nocebo effect. In previously seizure-free patients, switching the manufacturer of AED was associated with higher risk for seizure recurrence, with the authors suggesting a probable nocebo effect, under the impression that the replacement was motivated by cost reduction (Lang et al., 2018). Therefore, commercial features of a drug, such as the price, may have a strong impact on medication efficacy, through patient’s expectations (Waber et al., 2008). Furthermore, drug labeling with a known product name or pharmaceutical company name gives credence to its authenticity, efficacy, and scientific research and becomes more powerful as physicians refer to most medications by their brand name (Steinman et al., 2007). We found an fMRI study (Fehse et al., 2015), which demonstrated a behavioral placebo response only for the original brand, and also that brand labeling was associated with increased activity in brain areas that are known to be activated in the placebo analgesia. Also, a clinical study on headache investigating the impact of drug labeling on medication effectiveness and safety (Faasse et al., 2016) revealed that branded tablets (active or placebo) had similar effectiveness and that generic labeled placebo had significantly higher AEs than brand-name labeled placebo. Moreover, a retrospective study (Ngo et al., 2013) demonstrated that the majority of the patients were uncomfortable taking a generic AED, while the majority of them would use generics for acute short-term conditions, like painkillers. We could speculate that in situations where participants need to take medications for longer time periods to treat serious conditions, the effect of branding may be enhanced.

Finally, nocebo effect can be triggered by external factors such as the color and the shape of a pill (de Craen et al., 1996). Controversial, however, were the results of two studies concerning the risk of seizure when switching the manufacturer with emphasis to possible differences concerning the shape and color of the innovator and its generic product. The most prominent difference in those studies was the age of the participants, with younger patients not having an increased risk for seizures when switching generic’s manufacturer (Kesselheim et al., 2016) and older patients demonstrating a positive association between seizure incidence and change of manufacturer (40% of them older than 60 years) (Lang et al., 2018). Elderly patients are frequently on polypharmacy and present a variety of comorbidities, and changes in drugs’ shape and color may confuse them and increase their anxiety. To a certain degree, we could assume an interference of a possible nocebo effect, through negative expectancies resulting in non-adherence.

Bearing in mind the aforementioned, a tendency of higher incidence of nocebo effect concerning biosimilar and generic substitution in neurological diseases could be assumed in open-label and real-world studies compared to RCTs. A possible explanation could be the fact that patients who are skeptical of trying a new drug would not participate in RCTs, and consequently, such patients might be more highly included in open-label and real-world studies (Mitsikostas, 2012). Also, patients in the real world are more prone to experience nocebo effects because of previous treatment negative experiences conditioning (Kravvariti et al., 2018). Furthermore, methodological issues among RCTs and open-label or real-world studies could be of significance for the emergence of nocebo phenomenon. Frequently, investigators in RCTs through very close follow-up try to calm down and convince participants to continue the treatment in order to reduce the dropout ratios (Mitsikostas et al., 2012). Also, RCTs by default avoid the concerns of investigators expectations (positive or negative), while in open-label or real-world studies, investigators’ reporting may be driven by their preconceptions (Kravvariti et al., 2018). Especially for autoimmune inflammatory diseases, like MS, spontaneous relapses and exacerbations characterize their course, even in patients receiving treatment, making it difficult to differentiate from medication inefficacy. In RCTs, investigators, to overcome these temporal variations in disease activity, use run-in periods, which is something that is not feasible in real-world studies (Rief et al., 2017). Consequently, studying the “real” reason behind the referenced “adverse event” or “dropout due to adverse event,” and to what extent a contribution of a true nocebo response or effect is present, appears mandatory. Besides, reduced efficacy or tolerability might be related to other conditions except from nocebo. For example, neurological diseases, such as epilepsy, may have an unpredictable course and the timing of reported AEs may be coincidental and not causally related to the switch to a generic medication (Privitera et al., 2016). Pharmacokinetic factors could also participate, as in the case of biosimilars, where intrinsic differences in the drug molecules could not be excluded (Declerck et al., 2017). Interestingly, the GATE trial demonstrated that immediate post-injection adverse events were higher in the generic group when compared to the brand group (6.5 *vs.* 5.0%), whereas in the placebo group, such reactions did not occur. (Rezk and Pieper, 2017). Even though the above ratio was not statistically significant, the difference in vehicle or medium of the generic drug that potentially is more irritating to the skin could be a possible explanation. Nevertheless, it is unsafe to make this assumption and to extract results regarding the real drug-related adverse events that are attributed to the difference in excipients or even to minor impurities. The optimal scenario for detecting those “real” generic-related adverse events, are double-blind, randomized studies with placebo arm, ideally when different types of AEs between originator and bioequivalent/biosimilar arms occur, so that the aforementioned hypothesis would then be more solid. Also, particularly for AEDs, they share as a group numerous pharmacokinetic factors that may increase the probability of problems associated with generic substitution (Crawford et al., 2006). Carbamazepine, sodium valproate, and PHT are considered drugs of narrow therapeutic index, “where small differences in dose or blood concentration may lead to serious therapeutic failures and/or adverse drug reactions” (Yu et al., 2015). For LTG, two RCTs exist, with a duration of 2 months, which demonstrated bioequivalence between brand and generic LTG (Ting et al., 2015) and between two different generic LTG products (Privitera et al., 2016). In the case of LEV, two open-label bioequivalence studies exist (Markoula et al., 2017; Reimers et al., 2017), with a duration of 4 and 8 weeks, respectively, where equal fluctuation of LEV serum concentrations with branded and generic products was found. Nevertheless, under everyday clinical conditions, over longer time and where adhesion issues may occur, generic product could not be excluded to exhibit larger fluctuations in serum concentrations compared to the original product, at least in individual patients. Also, the refilling process itself may be associated with the increased frequency of seizures irrespective of whether refilling involved the same generic or different generic AEDs that could be attributed to minor but important changes in bioavailability related to the refilling process (Kesselheim et al., 2016). Finally, drug interactions may play a crucial role in efficacy and tolerability, especially for epileptic patients who often require polytherapy and can be sensitive to slight variations in drug bioavailability that may occur with generic medicines (Chaluvadi et al., 2011).

To that direction, altering trials’ design for better discrimination of nocebo is obligatory. The ideal trial design for nocebo discrimination from the spontaneous variations in the disease activity should include three arms (experimental drug/placebo/no drug), but this design is unethical and thus impossible for neurologic populations (Colloca and Miller, 2011), and consequently, other approaches could be adopted. A crossover design, in which each patient serves as its own control and the confounding effects of patient-related susceptibility are neutralized, could be an alternative approach (Kravvariti et al., 2018). Furthermore, incorporation of clinical tools for stratification of “high-risk” patients for nocebo, where their reported AEs could be interpreted accordingly, is highly suggested (Faasse and Petrie, 2013).

Particularly for neurological patients, Q-No is a four-item self-report questionnaire for outpatients seeking neurological consultation developed to predict the risk of nocebo (Mitsikostas and Deligianni, 2015). Another useful tool could be the General Assessment of Side Effects Scale, consisting of 36 items asking for symptoms of all body parts during the last 7 days. After rating the single symptom, the patient has to decide whether the symptom is related to the current medication, contributing to discriminate whether symptoms are really drug-induced or whether symptoms pre-existed (Rief et al., 2011). Additionally, the words in consent forms should be carefully selected and balanced between the expected benefits and AEs, inspiring confidence toward shared decision-making (Howick, 2012).

Additionally, every clinician should adopt several strategies for minimizing nocebo in neurological patients treated with biosimilars and generics. Firstly, as mentioned before, given the significant knowledge gap about generics and biosimilars in neurology, it is mandatory that all clinicians should be well informed about these medications, before prescribing them. Doctors, who are confident about a drug’s efficacy and safety, are able to transmit their confidence to the patients both with verbal and non-verbal positive suggestions (Rezk and Pieper, 2017). Recognizing also the patients at risk for being nocebo responders is crucial, using several clinical tools (Faasse and Petrie, 2013). Except from the Q-No questionnaire (Mitsikostas and Deligianni, 2015), other useful scales are the 10-item Beliefs about Medicines Questionnaire, which measures attitudes toward medication in general and medication prescribed for personal use (Horne et al., 1999), as well as the Perceived Sensitivity to Medicines Scale investigating quickly patient’s concerns about drug sensitivity (Horne et al., 2013).

Patient–clinician and patient–pharmacist relationship is of greatest importance in order to diminish the nocebo effect and enhance the placebo effect (Klinger et al., 2017), and the strategy of informed shared decision-making is highly encouraged (Rezk and Pieper, 2018). More specifically, clinicians should avoid using negative verbal suggestions and are encouraged to adopt a positive framing in their discussions with the patients in order to negate negative expectancies (Klinger et al., 2017). Doctors should explain the mechanisms of action of a medication and emphasize its positive outcomes regarding efficacy, without overemphasize its side effects. The choice of words is crucial and should not be confused with withholding negative information (Wells and Kaptchuk, 2012). For example, for biosimilars switching, the clinician could emphasize the equality and safety of the treatment to originator biologic instead of overemphasizing the remote chance of a small difference with unknown clinical consequence (Kristensen et al., 2018). For patients at risk of developing a nocebo effect, it is highly recommended that clinicians should familiarize them with the term and its consequences to their treatment, trying to provide them with the appropriate time to ask about the nocebo and possible negative aspects of the current therapy, dissolving any misconceptions (Klinger et al., 2017). Finally, when patients have had negative conditioning from previous therapies, clinicians should encourage them to describe their negative memories and take them very seriously into account, trying to demonstrate empathy (Colloca and Finniss, 2012).

Several limitations which compromised their external validity were identified in the included studies, mainly by their authors. Firstly, studies were conducted in different countries settings, where differences exist concerning prescription, dispensation, reimbursement of generics, and freedom to switchback, a fact that could have influenced certain results (Chaluvadi et al., 2011; Bosnyak et al., 2014; Piguet et al., 2015; Gollwitzer et al., 2016; Reimers et al., 2017; Lang et al., 2018). Furthermore, in several studies, data collection was retrospective (Crawford et al., 1996; Guberman and Corman, 2000; Wilner, 2002; Berg et al., 2008a; Berg et al., 2008b; Duh et al., 2009; Chaluvadi et al., 2011; Ngo et al., 2013; Gagne et al., 2015; Gollwitzer et al., 2016; Kesselheim et al., 2016; Roth et al., 2016; Bosak et al., 2017; Rahman et al., 2017; Gha-Hyun and Dae, 2018; Lang et al., 2018); thus, an ascertainment bias could not be excluded. As for the study populations, varying sample sizes were identified. Several authors stated, as a limitation of their study, the limited number of patients included (Mikati et al., 1992; Guberman and Corman, 2000; Bautista et al., 2011; Abolfazli et al., 2012; Ngo et al., 2013; Fehse et al., 2015; Piguet et al., 2015; Ting et al., 2015; Faasse et al., 2016; Privitera et al., 2016; Vari et al., 2016; Bosak et al., 2017; Fanella et al., 2017; Markoula et al., 2017; Gha-Hyun and Dae, 2018; Saposnik et al., 2018), while two large population-based studies (Gollwitzer et al., 2016; Kesselheim et al., 2016) were included. Moreover, in five studies, patients’ recruitment was made from a tertiary epilepsy clinic (Guberman and Corman, 2000; Bautista et al., 2011; Bosak et al., 2017; Das et al., 2019; Gha-Hyun and Dae, 2018), thus making the generalization of their results difficult. On the contrary, Gollwitzer et al., (2016) and Lang et al., (2018) stated as a limitation missing data from patients being treated in outpatient departments of hospitals, and also, Kesselheim et al., (2016) reported that patients who experienced seizures that did not require hospital visits or medical care were not included to their results. Studies’ outcomes derived from populations with different clinical characteristics, demanding also a careful interpretation of their results. Indicatively, age groups differed significantly in three studies, mainly young population in one of them (Kesselheim et al., 2016), and mainly elderly on the two others (Gagne et al., 2015; Lang et al., 2018). For studies on epilepsy, differences were observed among the studied type of epileptic syndromes (Privitera et al., 2016) (Fanella et al., 2017; Markoula et al., 2017; Trimboli et al., 2018), while in four studies based on database data (Duh et al., 2009; Gagne et al., 2015; Gollwitzer et al., 2016; Rahman et al., 2017), inaccuracies in coding of diagnosis could not been ruled out. Heterogeneity was observed also among studied populations regarding whether being on AED monotherapy or polytherapy, with studies mainly homogeneous (Bosak et al., 2017; Fanella et al., 2017; Trimboli et al., 2018) and others more heterogeneous (Bautista et al., 2011; Vari et al., 2016; Markoula et al., 2017). Additionally, a selection bias could not be excluded in several studies for a variety of reasons. Firstly, due to study design, in open-label studies, patients’ attitudes toward generics may have influenced their results, as frequently patients who were negative about generics were excluded (Ngo et al., 2013; Bosnyak et al., 2014; Vari et al., 2016; Markoula et al., 2017; Reimers et al., 2017; Gha-Hyun and Dae, 2018; Trimboli et al., 2018). Nevertheless, two studies focused on generic brittle patients (Ting et al., 2015; Das et al., 2018); in one of them, however, the assessment of generic sensitivity was mainly based on patients’ opinions (Das et al., 2018) and, also in another study, generic brittle patients constituted only a minority among study’s population (Privitera et al., 2016). Secondly, Berg et al., (2008b) reported a possible selection bias due to the online nature of their study and Roth et al., (2016) due to the fact that the pharmacists included in their study were those who attended meetings and scientific courses and thus were more knowledgeable about generics that others. Furthermore, several confounding factors that might have influenced studies’ results were identified by their authors. For example, several studies reported lack of information on variables that could have influenced seizure control, mainly because they were based on self-reported data (Mikati et al., 1992; Berg et al., 2008b; Bautista et al., 2011; Markoula et al., 2017; Gha-Hyun and Dae, 2018; Trimboli et al., 2018) or data extracted from databases (Gagne et al., 2015; Kesselheim et al., 2016). Indicatively, a limitation on adherence documentation was recognized in many studies (Mikati et al., 1992; Bautista et al., 2011; Trimboli et al., 2018). However, one study included a strict protocol for adherence and seizure documentation (Privitera et al., 2016), while another one excluded from analysis possible confounders that could contribute to breakthrough seizures (Berg et al., 2008a), and finally, another one study used a widely adopted measure of adherence in population-based studies (Gollwitzer et al., 2016). Measured clinical outcomes might need careful interpretation, as in some studies are based on self-reported data (Berg et al., 2008b; Das et al., 2018; Lang et al., 2018) or may be affected by external factors such as mass media advertisements (Rahman et al., 2017), or healthy policy and restrictive prescription rules (Saposnik et al., 2018), while some studies did not provide any data on AEs (Abolfazli et al., 2012; Reimers et al., 2017; Gha-Hyun and Dae, 2018). Many studies lacked pharmacokinetic data (Bosnyak et al., 2014; Vari et al., 2016; Bosak et al., 2017; Fanella et al., 2017; Gha-Hyun and Dae, 2018; Trimboli et al., 2018), while others provided (Ting et al., 2015; Privitera et al., 2016; Markoula et al., 2017; Reimers et al., 2017). Also, study’s duration was claimed short in three RCTs (Cohen et al., 2015; Ting et al., 2015; Privitera et al., 2016), in a retrospective study (Chaluvadi et al., 2011), and in two prospective studies (Abolfazli et al., 2012; Fanella et al., 2017). Finally, certain studies focused on a certain AED or a certain formulation of one AED (Ting et al., 2015; Privitera et al., 2016; Reimers et al., 2017) or dopamine agonist (Bosnyak et al., 2014).

Notwithstanding, this systematic review is not without its limitations. We only searched the MEDLINE database, and consequently, there is always the possibility of publication bias. Further, we did not perform a meta-analysis because of the heterogeneity of the study designs (RCTs, cohort studies, cross-sectional and case–control studies), populations, and cofactors. A strength of this review is that it was conducted with a systematic strategy, trying to shed light on a scientific query that has not been studied clearly, trying to suggest strategies for clinical trials design and clinical practice in order to investigate and minimize nocebo behavior in generics and biosimilars era in neurology.

## Conclusion

Nocebo represents a complex neurobiological behavior that is partly driven by patient’s conditioning by previous experiences and negative expectations and affects treatment outcomes and adherence significantly. In the era of generic and biosimilar medicine in particular, nocebo represents a real clinical and scientific challenge. This systematic review among the most common neurological conditions confirms that the true burden of the nocebo response in generic and biosimilar agents and effect cannot be estimated because the existing studies are not placebo controlled. However, nocebo as a phenomenon does exist and a growing percentage of investigators recognize that it may partially influence generics’ and biosimilars’ efficacy and tolerability. It hence lays the foundation for future studies design of biosimilars and generics in neurology, targeting to nocebo measurement either by using placebo arms or specific questionnaires predicting nocebo, like the Q-No questionnaire. Additionally, the expecting presence of nocebo in practice suggests individual strategies to identify the “high-risk” patients and treat them accordingly in order to improve good outcomes.

## Author Contributions

Conception and design: DM. Acquisition of data: IS, TM, DM. Analysis and interpretation of data: IS, TM, DM. Drafting the manuscript: IS. Revising it for intellectual content: IS, TM, DM. Final approval of the completed manuscript: IS, TM, DM.

## Conflict of Interest Statement

The authors declare that the research was conducted in the absence of any commercial or financial relationships that could be construed as a potential conflict of interest.
